# The effectiveness of thoracic medial branch radiofrequency ablation using a three-tined electrode: A real-world cross-sectional cohort study

**DOI:** 10.1016/j.inpm.2025.100649

**Published:** 2025-10-31

**Authors:** Hasan Sen, Edvin Koshi, Matthew Essman, Amanda N. Cooper, Audrey Adler, Akbar Nabi, Blake Dickenson, William Tang, Chase Young, Taylor Burnham, Alexandra E. Fogarty, Allison Glinka Przybysz, Aaron M. Conger, Zachary L. McCormick

**Affiliations:** a Department of Physical Medicine and Rehabilitation, University of Utah, Salt Lake City, UT, United States; b Department of Physical Medicine and Rehabilitation, Dalhousie University, Halifax, Nova Scotia, Canada; c Vivo Cura Health, Calgary, Alberta, Canada

**Keywords:** Radiofrequency ablation, Thoracic medial branch, Facet joint pain

## Abstract

**Background:**

Thoracic medial branch radiofrequency ablation (TMBRFA) is used to treat chronic thoracic spine pain, but data regarding its effectiveness and technique remain limited.

**Objectives:**

Evaluate outcomes of TMBRFA using a three-tined electrode in patients with thoracic facet joint pain.

**Methods:**

Consecutive patients who underwent first-time TMBRFA at a single tertiary academic center between 2022 and 2024 were identified and contacted for follow-up via standardized telephone survey at ≥6 months post-procedure. Baseline demographic and clinical data were extracted from electronic medical records. Outcomes included the proportions of patients with ≥50% numerical rating scale (NRS) pain score reduction, ≥2-point NRS reduction, and Patient Global Impression of Change (PGIC) scores ≥6 (indicating at least “much improved”). Mean NRS pain score reduction and changes in opioid use from baseline were also assessed.

**Results:**

Outcomes were successfully collected from 11 patients. At a mean follow-up of 20.6 ± 7.6 months, 36.4% (95%CI: 15.2–64.6) and 45.5% (95%CI: 21.3–72.0) of patients reported ≥50% and ≥2-point NRS reductions, respectively, with 54.6% (95%CI: 28.0–78.7) reporting PGIC scores ≥6. Mean NRS pain score reduction was 1.9 ± 2.7 points. Two patients had ceased using opioids at follow-up, representing a proportional decrease of 18.2% compared to baseline.

**Conclusion:**

In this cohort, approximately half of patients who underwent first-time TMBRFA with a three-tined electrode experienced clinically meaningful pain relief and perceived overall improvement at an average follow-up of nearly 2 years. Larger, prospective studies are needed to corroborate these findings.

## 1 Introduction

Thoracic spine pain is less commonly encountered than pain originating from other spinal regions, constituting approximately 5% of patient referrals to outpatient pain clinics [1]. As a result, thoracic spine pain research has historically been neglected compared to the cervical and lumbar spine. This includes research related to the treatment of thoracic zygapophyseal “facet” joint pain.

The International Pain and Spine Intervention Society (IPSIS) Practice Guidelines [2] outline a technique for thoracic medial branch (TMB) blocks to establish a diagnosis of thoracic facet joint pain based on historical dissection study [3]. However, the guidelines do not describe or recommend a specific technique for thoracic medial branch radiofrequency ablation (TMBRFA). Notably, more recent dissections indicate that thoracic facet joint innervation may vary and be more complex than originally described by Chua and Bogduk [[2], [3], [4]], but early evidence suggests that favorable clinical outcomes are observed when utilizing the IPSIS guidelines landmarks [[5], [6], [7], [8]].

Two separate techniques for TMBRFA using conventional radiofrequency ablation (RFA) electrodes have been described to date by Hambraeus and Derby et al. [5,6]. In 2018, Hambraeus et al. [5] described a TMBRFA protocol using a conventional 18-gauge RF needle with a 10-mm active tip heated to 80°C to create a series of three to 15 lesions lasting 60 s each along the superior border of the transverse process. A second protocol was proposed by Derby et al. [6], who described one and two-needle techniques using parallel cannulae placed 5–10 mm apart. Derby et al. utilized a 75-degree medial-to-lateral approach, inserting the needle at an angle approximately 15–30° to the skin with an entry point midway between the transverse processes. An angle of approach as described by Derby et al. is necessary when using a conventional RFA electrode given that the lesion largely forms adjacent to the long axis of the active tip. This creates a technical challenge.

More recently, novel technologies have been developed which allow for a perpendicular approach to target nerves during radiofrequency ablation procedures. The Diros Trident™ RF cannula is a three-tined electrode that creates a relatively large distal lesion while maintaining a smaller proximal footprint. The greatest surface area of the lesion is generated at the distal aspect of the three tines, with a tapered geometry to the lesion more proximally. This pyramidal lesion morphology results in effective target nerve capture while minimizing more superficial tissue coagulation when the electrode is oriented perpendicular to the nerve. This approach confers technical advantages and may improve patient comfort by reducing the length of tissue that must be traversed to reach the intended target(s). Because the size and shape of the lesion do not change with the probe angle [9], an appropriate perpendicular approach to the transverse process may be achieved when TMBRFA is performed utilizing the IPSIS Practice Guidelines technique for targeting thoracic medial branches.

To our knowledge, only one study to date has reported outcomes of TMBRFA using a multi-tined Trident electrode with a perpendicular approach to the transverse process. Sen et al. found that just over 55% of patients treated with this technique reported ≥50% pain reduction at 3 months post-procedure in a cohort of patients in Canada [10]. These early results highlight the therapeutic potential of TMBRFA when multi-tined electrodes are used to target thoracic medial branch nerves according to standard historical landmarks [2,3].

In the present study, we aimed to evaluate the effectiveness of TMBRFA in a real-world population using a three-tined electrode by assessing patient-reported outcomes at longer-term follow-up than has been previously reported.

## 2 Methods

### 2.1 Data collection

This cross-sectional cohort study was conducted at a tertiary academic medical center. Local Institutional Review Board (IRB 00187326) approval was obtained. The electronic medical records of consecutive patients who underwent first-time TMBRFA between 2022 and 2024 were reviewed. Inclusion criteria were (1) age ≥18 years old; (2) ≥80% pain relief with dual, concordant medial branch nerve blocks (MBBs) to confirm a diagnosis of thoracic facet pain; (3) TMBRFA procedure performed with three-tined RFA electrode technology; (4) thoracic spine pain severity score ≥4/10 on Numeric Rating Scale (NRS) documented within 2 months prior to TMBRFA procedure; (5) ≥6 months between TMBRFA and follow-up outcomes survey; and (6) willingness to participate in a post-procedural phone call survey. Data extraction was performed by the authors (HS, WT, ANC). Data collected included the following: (1) age; (2) gender; (3) body mass index (BMI); (4) current smoking status; (5) duration of thoracic pain prior to TMBRFA in years; (6) baseline daily anxiety or depression medication use; (7) baseline opioid use; (8) history of thoracic spinal fusion; (9) highest recorded NRS thoracic pain score within two months before TMBRFA; (10) percentage of pain improvement with each MBB; (11) date of TMBRFA; and (12) treatment level(s) and laterality. Patients who met the inclusion criteria were contacted via a letter sent on behalf of their treating physician regarding the study. Those who agreed to participate completed a standardized post-TMBRFA phone call survey, which captured current and 7-day average NRS pain scores, self-reported improvement by the Patient Global Impression of Change (PGIC), and use of opioid analgesics to manage their index thoracic pain.

### 2.2 Procedures

#### 2.2.1 Radiofrequency ablation

**Fig. 1:**
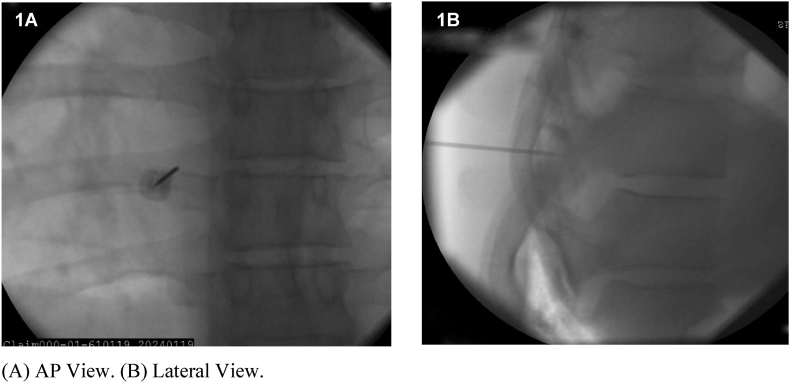
Goal electrode position for the left T7 medial branch.

**Fig. 2:**
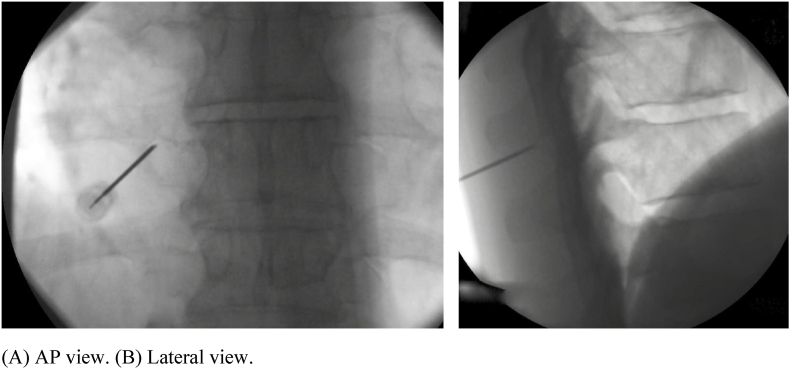
Goal electrode position for the left T9 medial branch.

**Fig. 3:**
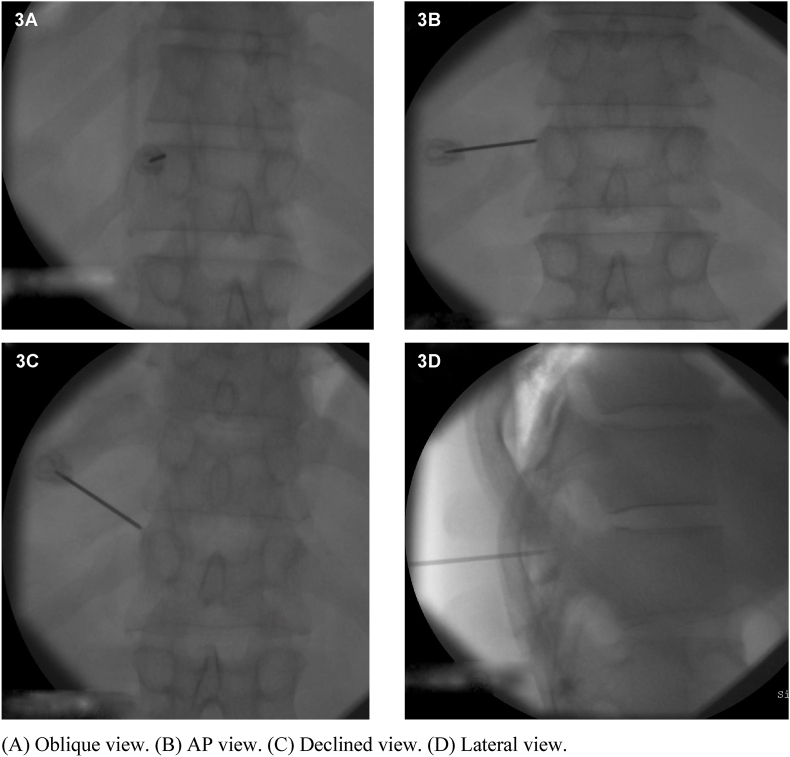
Goal electrode position for the left T11 medial branch.

**Fig. 4:**
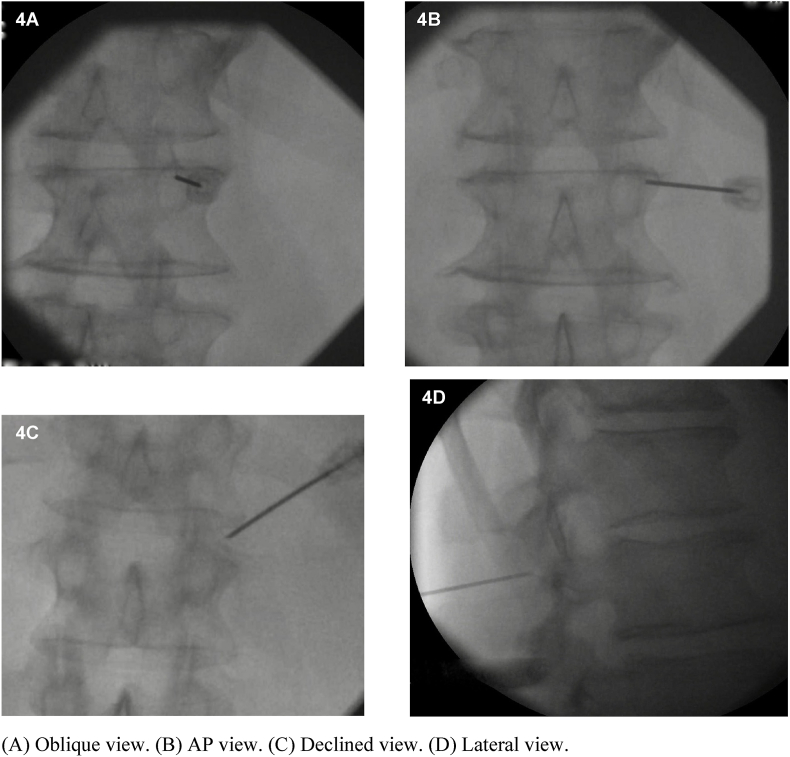
Goal electrode position for the right T12 medial branch.

All TMBRFA procedures were performed by Physical Medicine and Rehabilitation physician faculty members with fellowship training in Interventional Spine & Musculoskeletal Medicine or Pain Medicine. These procedures were performed in an identical fashion to that previously described [10], with the exception that intravenous midazolam and/or fentanyl (rather than sublingual lorazepam and/or inhaled nitrous oxide) were administered to patients for anxiolysis and/or pain control when medically indicated. RFA electrode placements were based on IPSIS guidelines describing anatomical landmarks for thoracic medial branch blocks [2,3]. Representative examples are shown in Fig. 1, Fig. 2, Fig. 3, Fig. 4. Monopolar RF lesions were created using generator settings of 2 min at 90°C with a 30-s ramp up time.

### 2.3 Outcome measurement

The primary outcome was the proportion of patients with ≥50% pain reduction at least six months after TMBRFA. Secondary outcomes included (1) the proportion of patients who reported ≥2-point NRS reduction from baseline, which is the minimal clinically important change (MCID) for cervical and lumbar spine pain (as an MCID for thoracic spine pain has yet to be defined) [11,12], (2) the mean change in NRS pain score from baseline, (3) the proportion of patients who reported being “much improved” or “very much improved” on PGIC (scores of 6–7), and (4) the proportion of patients using opioid analgesics at follow-up compared to baseline.

### 2.4 Statistical analysis

Participant demographics, clinical characteristics, and procedure-related variables were summarized using descriptive statistics, with calculations of frequencies/percentages for categorical variables and means/standard deviations for continuous variables. Additionally, 95% confidence intervals (CI) were calculated for categorical outcome variables. McNemar's test assessed for differences in opioid medication use at baseline and follow-up.

## 3 Results

Eleven patients (90.9% female; mean age 60.1 ± 16.6 years) with first-time TMBRFA procedures were included in the analysis. Baseline demographics and clinical characteristics are summarized in Table 1. The average time to follow-up was 20.6 ± 7.6 months, with outcomes collected at 6–12 months (*n* = 2; 18.2%), 12–18 months (*n* = 2; 18.2%), 18–24 months (*n* = 3; 27.3%), and ≥24 months (*n* = 4; 36.4%) post-procedure.

**Table 1:** Baseline participant demographics, clinical characteristics, and procedural variables.

Variable	No. (%)
Follow-up duration after TMBRFA	
6–12 months	2 (18.2)
12–18 months	2 (18.2)
18–24 months	3 (27.3)
≥24 months	4 (36.4)
Gender	
Male	1 (9.1)
Female	10 (90.9)
Current smoking status	
No	11 (100.0)
Yes	0 (0.0)
History of thoracic spinal fusion	
No	9 (81.8)
Yes	2 (18.2)
Daily anxiety/depression medication use	
No	4 (36.4)
Yes	7 (63.6)
Pain relief with first MBB	
80–99%	1 (9.1)
100%	10 (90.9)
Pain relief with second MBB	
80–99%	0 (0.0)
100%	11 (100.0)
TMBRFA laterality	
Unilateral	2 (18.2)
Bilateral	9 (81.8)

Age in years, mean ± SD	60.1 ± 16.6
BMI in kg/m^2^, mean ± SD	27.6 ± 5.1
Pain duration in years, mean ± SD	5.5 ± 5.4
Follow-up time in months, mean ± SD	20.6 ± 7.6

Note: Values are frequency (%), unless specified otherwise.

MBB = medial branch block; SD = standard deviation; TMBRFA = thoracic medial branch radiofrequency ablation.

Regarding outcomes for pain relief, 36.4% (*n* = 4; 95% CI: 15.2, 64.6) of patients reported ≥50% reduction in NRS pain scores, and 45.5% (*n* = 5; 95% CI: 21.3, 72.0) reported a ≥2-point reduction at a mean follow-up of 20.6 ± 7.6 months (Table 2). The mean change in NRS pain score from baseline was 1.9 ± 2.7 points at average follow-up.

**Table 2:** Outcomes for pain relief.

Outcome	No. (%)	95% CI
≥50% NRS reduction		
Yes	4 (36.4)	15.2, 64.6
No	7 (63.6)	35.4, 84.8

≥2-point NRS reduction		
Yes	5 (45.5)	21.3, 72.0
No	6 (54.6)	28.0, 78.7

NRS score reduction from baseline, mean ± SD	1.9 ± 2.7	0.1, 3.7

Note: Values are frequency (%), unless specified otherwise.

CI = confidence interval; NRS = Numerical Rating Scale; SD = standard deviation.

Patient Global Impression of Change (PGIC) scores ≥6, indicative of being “much” or “very much improved,” were reported by 54.6% (*n* = 6; 95% CI: 28.0, 78.7) of patients at mean follow-up (Table 3).

**Table 3:** Outcomes for patient global impression of change.

Outcome	No. (%)	95% CI
≥6 on PGIC		
Yes	6 (54.6)	28.0, 78.7
No	5 (45.5)	21.3, 72.0

Note: Values are frequency (%), unless specified otherwise.

CI = confidence interval; PGIC = Patient Global Impression of Change.

PGIC scores ≥6 indicate at least “much improved”.

In this cohort, the proportion of patients using opioid medications for pain management decreased from 36.4% (*n* = 4; 95% CI: 15.2, 64.6) at baseline to 18.2% (*n* = 2; 95% CI: 5.1, 47.7) at follow-up (Table 4a). However, this reduction was not statistically significant (McNemar's test; *p* = 0.500; Table 4b).

**Table 4a:** Opioid use at baseline and follow-up.

Opioid use	Yes	No	95% CI (yes)
Baseline	4 (36.4)	7 (63.6)	15.2, 64.6
Follow-up	2 (18.2)	9 (81.8)	5.1, 47.7

Note: Values are frequency (%), unless specified otherwise.

CI = confidence interval.

**Table 4b:** Changes in opioid use from baseline to follow-up.

Opioid use (baseline)	Opioid use (follow-up)			Difference in proportions (95% CI)
	Yes	No	P*	
Yes	2 (18.2)	2 (18.2)	0.500	18.2 (−13.7, 50.1)
No	0 (0.0)	7 (63.6)		

Note: Values are frequency (%), unless specified otherwise.

CI = confidence interval.

*From McNemar's exact test.

## 4 Discussion

In this cross-sectional cohort study of TMBRFA using a three-tined RFA electrode, 36.4% and 45.5% of participants reported ≥50% and ≥2-point NRS pain score reductions from baseline, respectively, at long-term follow-up. Similarly, 54.6% of patients reported PGIC scores of 6 or 7, indicating meaningful subjective improvement post-procedure. To our knowledge, only our recent retrospective study has reported clinical outcomes of TMBRFA using the Trident™ cannula with a perpendicular approach to date [10]. Outcomes for pain and quality of life at 3 months were similar to those seen in the present cohort, with 55.6% and 50.0% of patients reporting ≥50% NRS reduction and the MCID on the Pain Disability Quality-Of-Life Questionnaire-Spine (PDQQ-S), respectively [10]. The present findings indicate relative durability of improvement in pain at longer-term follow-up. This is encouraging given that this technique may improve patient comfort and reduce radiation exposure compared to previous TMBRFA protocols, though larger studies are needed to confirm these findings [13,14].

Our outcome findings fall within the range of prior reports investigating thoracic medial branch radiofrequency ablation. Rohof and Chen reported that 82% of patients treated with bipolar RFA achieved at least 50% pain relief at 12 months, potentially due to the larger lesions created by the bipolar technique [15]. Abd Elsayed et al. found a mean pain reduction of 62.8% at one year in 55 patients undergoing internally cooled RFA, with nearly 78% reporting improvement [16]. Conversely, Gungor and Candan observed more modest pain relief of 37.6% at 6–12 months in a smaller cohort treated with internally cooled RFA [17]. Together, these studies illustrate a range of outcomes potentially influenced by RFA technique, lesion size, and patient selection.

The success of TMBRFA may be impacted by the documented variability of thoracic facet joint innervation in the middle and lower thoracic spinal levels. IPSIS guidelines for anesthetic blockade of the T1 to T3 medial branches, as well as the T9 and T10 medial branches, recommend targeting just medial to the superolateral corner of the dorsal aspect of transverse process given the relatively constant relationship between the nerve and bony landmark at these levels [2]. The junction of the superior articular process and root of the transverse process is the target area recommended for targeting the T11 and T12 medial branches, based on shared similarities with the lumbar region with respect to sagittal facet alignment [2]. Because the T4 to T8 medial branches are suspended in the intertransverse space, no bony landmark currently serves as a target for these levels [2]. However, IPSIS guidelines suggest targeting immediately dorsal to the rib at the level of the transverse process cephalad to the level of interest [2].

It is notable that other investigators have observed differences in thoracic medial branch anatomy when compared to the historical study by Chua and Bogduk [3]. Joshi et al. [4] found that most zygapophyseal branches at T4 to T8 originate directly from the posterior ramus, near the intervertebral foramen, with few articular branches arising from the medial branches. These findings support other previous reports [18] and raise notable controversy regarding where to target the medial branches at these mid-thoracic levels. Additional variability in medial branch anatomy at the T10-12 levels has been described by Koutp et al. [19]. These investigators found that almost 30% of cadavers had medial branches which passed parallel but superior to the previously recognized course without contacting the superolateral corner of the transverse process. These findings highlight the need for further anatomical studies, as well as innovative techniques with potentially adapted targets to optimize procedural outcomes.

### 4.1 Limitations

This study has several limitations that must be acknowledged. The primary limitation of this study is the small sample size. Additionally, the cross-sectional study design cannot establish causality. The lack of a control or comparator group precludes direct comparisons of outcomes associated with alternate approaches to TMBRFA. Lastly, unaccounted-for variability in patient characteristics, such as previous treatments and medical comorbidities, may have influenced results by introducing confounding factors.

## 5 Conclusion

This study provides preliminary evidence of the long-term effectiveness of TMBRFA using a perpendicular approach with a three-tined electrode system. At a mean follow-up of nearly 21 months, clinically meaningful improvements were observed in a proportion of patients, with 36.4% achieving ≥50% pain reduction, 45.5% experiencing a 2-point NRS pain reduction, and 54.6% reporting PGIC scores consistent with “much improved” or better. Larger prospective studies are warranted to confirm the present findings.

## Funding

This work was supported by investigator-initiated grant funding from Avanos Medical.

## Declaration of competing interest

The authors declare the following financial interests/personal relationships which may be considered as potential competing interests:

Zachary L. McCormick reports financial support was provided by Avanos Medical Inc. Zachary L. McCormick reports a relationship with International Pain & Spine Intervention Society that includes: board membership. Zachary L. McCormick reports a relationship with Avanos Medical Inc that includes: consulting or advisory and funding grants. Zachary L. McCormick reports a relationship with 10.13039/100008497Boston Scientific Corporation that includes: funding grants. Zachary L. McCormick reports a relationship with Presidio Medical that includes: funding grants. Zachary L. McCormick reports a relationship with SAOL Therapeutics that includes: consulting or advisory and funding grants. Zachary L. McCormick reports a relationship with Spine Biopharma that includes: funding grants. Zachary L. McCormick reports a relationship with SPR Therapeutics Inc that includes: funding grants. Zachary L. McCormick reports a relationship with Stratus Medical that includes: funding grants. Zachary L. McCormick reports a relationship with Stryker that includes: consulting or advisory. Zachary L. McCormick reports a relationship with OrthoSon that includes: consulting or advisory. Alexandra Fogarty reports a relationship with International Pain & Spine Intervention Society that includes: board membership. Aaron Conger reports a relationship with Stratus Medical that includes: funding grants. If there are other authors, they declare that they have no known competing financial interests or personal relationships that could have appeared to influence the work reported in this paper.
